# The Effect of a New Sodium Bicarbonate Loading Regimen on Anaerobic Capacity and Wrestling Performance

**DOI:** 10.3390/nu10060697

**Published:** 2018-05-30

**Authors:** Krzysztof Durkalec-Michalski, Emilia Ewa Zawieja, Tomasz Podgórski, Bogna Ewa Zawieja, Patrycja Michałowska, Igor Łoniewski, Jan Jeszka

**Affiliations:** 1 Institute of Human Nutrition and Dietetics, Poznan University of Life Sciences, 60-624 Poznań, Poland; emilia.zawieja@gmail.com (E.E.Z.); pati1701@o2.pl (P.M.); jeszkaj@up.poznan.pl (J.J.); 2 Polish Wrestling Federation, 00-871 Warsaw, Poland; 3 Department of Biochemistry, University School of Physical Education in Poznan, 61-871 Poznań, Poland; podgorski@awf.poznan.pl; 4 Department of Mathematical and Statistical Methods, Poznań University of Life Sciences, 60-637 Poznań, Poland; bogna13@up.poznan.pl; 5 Department of Biochemistry and Human Nutrition, Pomeranian Medical University, 71-460 Szczecin, Poland; igorloniewski@sanum.com.pl

**Keywords:** alkalosis, combat sports, Wingate test, dummy test, buffering

## Abstract

Gastrointestinal side effects are the main problem with sodium bicarbonate (SB) use in sports. Therefore, our study assessed the effect of a new SB loading regimen on anaerobic capacity and wrestling performance. Fifty-eight wrestlers were randomized to either a progressive-dose regimen of up to 100 mg∙kg^−1^ of SB or a placebo for 10 days. Before and after treatment, athletes completed an exercise protocol that comprised, in sequence, the first Wingate, dummy throw, and second Wingate tests. Blood samples were taken pre- and post-exercise. No gastrointestinal side effects were reported during the study. After SB treatment, there were no significant improvements in the outcomes of the Wingate and dummy throw tests. The only index that significantly improved with SB, compared to the placebo (*p* = 0.0142), was the time-to-peak power in the second Wingate test, which decreased from 3.44 ± 1.98 to 2.35 ± 1.17 s. There were also no differences in blood lactate or glucose concentrations. In conclusion, although the new loading regimen eliminated gastrointestinal symptoms, the doses could have been too small to elicit additional improvements in anaerobic power and wrestling performance. However, shortening the time-to-peak power during fatigue may be particularly valuable and is one of the variables contributing to the final success of a combat sports athlete.

## 1. Introduction

Wrestling is a combat sport that includes explosive attacks and counterattacks that are executed repeatedly [1]. Because of the high-intensity character of wrestling, the main energy system involves glycolysis, leading to muscle acidification [2]. The anaerobic power is associated with success in wrestling by contributing to the attack and lifting of an opponent during offensive maneuvers, in addition to resisting the opponent’s attacks [3]. In the conditions of reduced oxygen availability, glycolysis results in the accumulation of lactate and hydrogen ions (H^+^) [4]. Lactate concentrations after a simulated wrestling combat rise to ~12.5 mmol·L^−1^ in elite athletes [2]. The accumulation of H^+^ causes acidification in the muscle that is associated, among other things, with muscle fatigue. Additionally, lower serum bicarbonate and a higher anion gap are linked to lower cardiorespiratory fitness [5]. Nevertheless, the mechanisms of muscle fatigue are still unclear. The role of acidification on muscle fatigue may result from: (1) competition of H^+^ with calcium ions for the troponin binding site, which impairs the ability of the contractile machinery to effectively operate; (2) inhibition of phosphocreatine resynthesis; (3) inhibition of key enzymes of the glycolytic pathway, such as glycogen phosphorylase and phosphofructokinase [6]; and (4) decreased mitochondrial energy production in muscle cells due to a reduced mitochondrial matrix-cell cytoplasm proton gradient [7]. Maintaining pH within the physiological range is pivotal for sustaining muscle contractility [8]. During high-intensity exercise, intramuscular acidity is regulated by intracellular, extracellular, and dynamic buffering systems [6]. Bicarbonate, in particular, is the major contributor to the buffering system in the blood because it has the ability to bind H^+^ [9]. Oral supplementation with sodium bicarbonate increases blood bicarbonate concentrations and results in blood alkalosis, leading to a greater efflux of H^+^ and lactate out of active muscles and into the circulation [10]. It has also been suggested that metabolic alkalosis in skeletal muscles results in the acceleration of glycogenolysis, increasing the reliance on muscle glycogen stores as fuel during exercise [11]. Sostaric et al. found that alkalization could reduce membrane depolarization caused by exercise, which may enhance exercise performance [12]. However, most importantly, sodium bicarbonate supplementation may improve athletic performance due to H^+^ buffering and La^−^ efflux from exercising muscle, which allows sustaining muscle contractility during intense exercise [10,13]. Thus, it is important to conduct further research in this area.

In a study of judo athletes, the ingestion of 0.3 g·kg^−1^ body weight of sodium bicarbonate 120 min before the beginning of exercise resulted in better performance in the Special Judo Fitness Test (SJFT), measured as the total number of throws [14]. The number of throws was increased in rounds 2 and 3, but was not altered in round 1. Thus, this study demonstrated that ingesting sodium bicarbonate may cause a significant improvement in judo-related performance, especially during the final stages of exercise when fatigue is evident [14]. In another study, Felippe et al. [15] assessed the effect of a separate or combined intake of caffeine and sodium bicarbonate on judo performance (three repeated bouts of SJFTs interspaced with 5 min rest). They found, however, that only the combined use of sodium bicarbonate and caffeine increased the total number of throws during three successive SJFTs. More throws with sodium bicarbonate alone were only found during the third SJFT, but overall performance was only improved when sodium bicarbonate was combined with caffeine [15]. In a study by Siegler and Hirscher [16], the intake of 0.3 g·kg^−1^ of sodium bicarbonate improved boxing performance and punch efficacy and resulted in an elevated blood buffering status prior to the boxing match, with the elevation being sustained throughout the four rounds of boxing. Furthermore, Krustrup et al. [17] showed that sodium bicarbonate improved high-intensity intermittent exercise performance in the Yo-Yo intermittent recovery test level 2 by 14% compared to a placebo. The improvement in performance was accompanied by elevated blood alkalosis and concentration of bicarbonate, while the rating of perceived exertion was lower during intense exercise after sodium bicarbonate supplementation [17]. 

The benefits to athletic performance with the use of sodium bicarbonate are well-documented [13]. A meta-analysis by Carr et al. [18] showed that the oral ingestion of sodium bicarbonate resulted in a moderate performance augmentation of 1.7% with a dose of ~0.3 g∙kg^−1^ in a single 1-min sprint, with a further ~1% improvement in repeated sprint performance. The favorable effect of sodium bicarbonate supplementation on physical performance was also confirmed by Peart et al. [19] in a meta-analysis of 40 randomized clinical trials including 395 subjects. However, in a meta-analysis by Peart et al. [19], the beneficial ergogenic effect of sodium bicarbonate was only reported in 38% of analyzed articles. Nevertheless, sodium bicarbonate is recommended by International Olympic Committee (IOC) experts to be used by high-performance athletes [20]. IOC recommends the use of a single acute NaHCO_3_ dose of 0.2–0.4 g∙kg^−1^ body mass (BM), 60–150 min. prior to exercise or to split the same daily dose taken over the time period of 30–180 min. Another strategy is the serial intake of three to four smaller doses daily for two to four consecutive days prior to a competition. The ergogenic effect of sodium bicarbonate seems to be dose dependent [21]. However, the lowest effective dose of sodium bicarbonate is still controversial. In most studies, doses recommended by IOC are used. Horswill et al. [22] reported that doses of 200 mg∙kg ^−1^ and below resulted in an incremental increase in blood bicarbonate level, but did not influence performance. On the contrary, Browman showed [23] a beneficial effect of using 100 mg∙kg^−1^ sodium bicarbonate 60 min before time trial tests in swimmers.

However, the major limitation to the intake of higher doses of sodium bicarbonate is its gastrointestinal side effects, i.e., nausea, diarrhea, bloating, and thirst [23,24]. Saunders et al. [25] showed that athletes experiencing gastrointestinal distress are less likely to improve with sodium bicarbonate treatment. Individualized modifications to bicarbonate supplementation protocols can help prevent adverse effects [19]. To address this problem, we proposed a chronic, progressive-dose, sodium bicarbonate loading regimen. We aimed to examine the effect of sodium bicarbonate loading on performance using the Wingate cycling test and dummy throw tests.

## 2. Materials and Methods 

### 2.1. Participants

**Figure 1:**
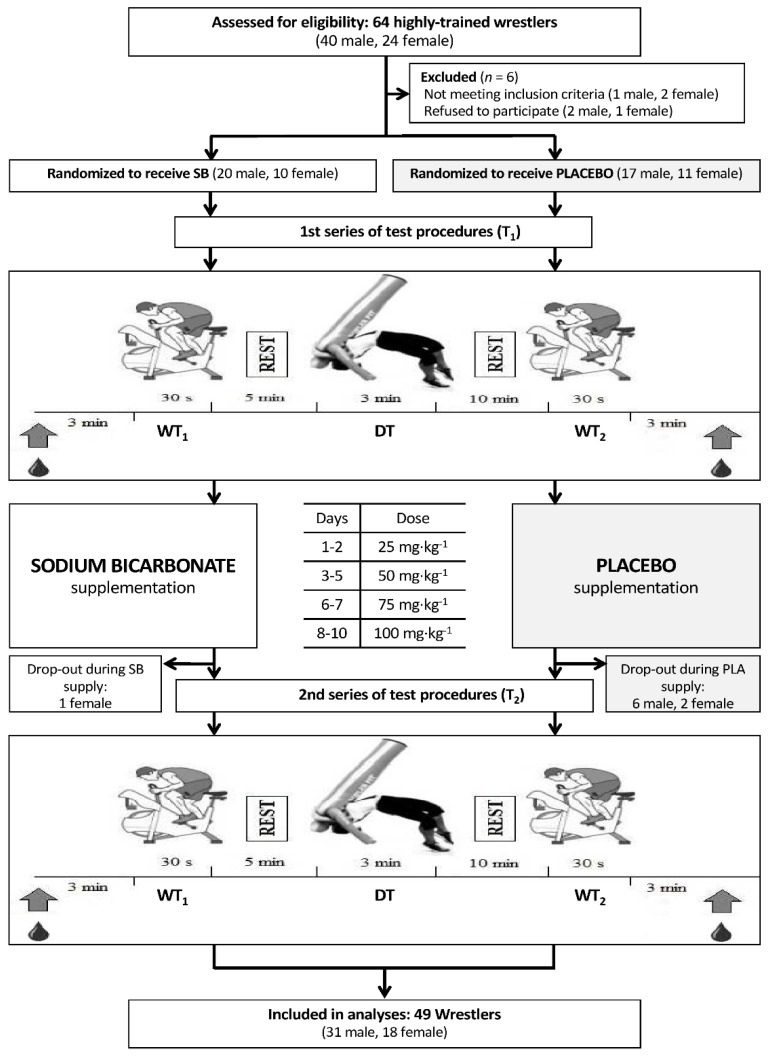
A flowchart of the study design. Abbreviations: T_1_—1st series of test procedures, T_2_—2nd series of test procedures, REST—the rest interval between exercise sets, PLA—placebo, SB—sodium bicarbonate, WT_1_—the Wingate test before the dummy throw test, WT_2_—the Wingate test after the dummy throw test, DT—the dummy throw test.

Forty-nine athletes (18 women, 31 men) completed the entire study protocol and were included in the analyses (Figure 1, Table 1). The athletes were members of the Polish Wrestling National Team and/or top wrestlers in national competitions. The inclusion criteria were a good condition of health, a valid and up-to-date medical certificate confirming the athlete’s ability to practice sports, at least four years of training experience, and participation in a minimum of four workout sessions (combat sport) a week. The exclusion criteria were current injury, any health-related contraindication, a declared general feeling of being unwell, and unwillingness to follow the study protocol. The drop-out rate was higher in the placebo group (Figure 1). However, it was not connected with the study protocol. The reasons for drop outs were minor injuries during the customary training practice, which prevented the athletes from participating in exercise tests, and personal reasons.

The studies were conducted from January 2015 to April 2015. No changes in lifestyle, training regimen, diet, or supplementation were allowed during the study, and athletes declared that they had not used any medications and supplements with potential ergogenic effects, other than those supplied in the present study. In accordance with the 1975 Declaration of Helsinki, all the participants consented to participate in the research procedures before the study began. Informed consent was also obtained from the parents of athletes under the age of 18 years, prior to participation in the study. The approval of the Bioethics Committee at Poznań University of Medical Sciences was obtained for this study. This trial was registered at Clinical Trials Gov (website: https://clinicaltrials.gov/ct2/show/NCT03406065; Clinical Trial Identification Number: NCT03406065). The study was registered retrospectively as registration was not required when the study enrolment started. The authors confirm that all ongoing and related trials associated with this intervention are registered. The study complies with the CONSORT statement for randomized trials, as shown in Figure 1 and Table S1.

### 2.2. Experimental Protocol

The study adopted a randomized double-blind placebo-controlled parallel-group design (Figure 1). The participants were intimated on the testing procedures, protocols, and equipment before the beginning of the study. Anthropometric measurements were taken on the preliminary visit. Upon qualification for the experiment, the athletes (matched on their lean body mass) were randomized either to a group receiving sodium bicarbonate (SB group) or to another group receiving a placebo (PLA group). The random allocation sequence and matching were performed using stratified randomization via impartial biostatistics. The primary outcome in our study was change in anaerobic capacity and a specific wrestler’s performance capacity. Lactate and glucose concentration were defined as secondary outcomes.

The main study protocol involved two other visits (T_1–2_) and included exercise tests conducted in natural conditions at the Sobieski Wrestling Training Center (Poznań, Poland). During the trials, the participants ingested either sodium bicarbonate or the placebo (maltodextrin and NaCl). The participants performed the exercise tests before and after each trial. All testing was performed at the same time of day. Blood samples were taken before and after each exercise test in order to measure blood lactate and glucose concentrations. The participants were instructed to arrive for testing sessions between 7.30 and 10.00 a.m. and to avoid strenuous exercise for the 24 h preceding each test session. 

In order to assess dietary intake, food diaries were given to the participants to record food and fluid consumption for five days. The participants also recorded training loads during both trials in the training diaries. The participants were asked to maintain the same dietary intake and training load throughout the study protocol. The analysis of dietary and workout records showed that the athletes in the studied groups did not differ in terms of dietary habits and training specificity during the treatment period.

### 2.3. Supplementation

For the sodium bicarbonate trial, the participants ingested sodium bicarbonate using a progressive-dose regimen in order to reduce the likelihood of gastrointestinal side effects. The dose of sodium bicarbonate was, on day 1–2, 25 mg∙kg^−1^ (25% of the final dose of 100 mg∙kg^−1^); on day 3–5, 50 mg∙kg^−1^ (50% of the final dose); on day 6–7, 75 mg∙kg^−1^ (75% of the final dose); and on day 8–10, 100 mg∙kg^−1^ (100% of the final dose). Sodium bicarbonate was administered in the form of unmarked disc-shaped tablets (Alkala T, manufacturer—Sanum Kehlbeck GmbH & Co. KG, Hoya, Germany). The tablets were ingested with at least 250 mL of water and could either be swallowed or dissolved in the mouth. In the PLA group, the participants ingested maltodextrin with NaCl in a similar tablet form prepared by the same producer of the sodium bicarbonate tablets. Both the sodium bicarbonate and placebo were ingested in three evenly split doses throughout the day. On the training days, the supplements were taken in the morning, in the evening, and 1.5 h before a training session. On rest days, the supplements were taken in the morning, in the afternoon, and in the evening. The participants were also provided with individually adjusted supplementation schedules. Regarding the double blinding, neither the researchers nor the participants knew whether sodium bicarbonate or the placebo was administered. Only the head of the department had access to the randomization information, which was only revealed after the cessation of the protocol. 

### 2.4. Anthropometric Measurements

At the preliminary visit to the laboratory, anthropometric measurements were taken with the participants in a fasted state during the morning hours. Body mass and height were measured using a professional medical scale with a stadiometer (WPT 60/150 OW, RADWAG^®^, Radom, Poland). The stadiometer had an accuracy of 0.1 cm and 0.1 kg for height and body mass, respectively. Body fat and free-fat mass were assessed based on air displacement plethysmography using the Bod Pod^®^ (Bod Pod^®^, Cosmed, Rome, Italy). Once the body density was determined, the body fat and free-fat mass were calculated using the Siri equation. Thoracic lung volume was estimated using the Bod Pod^®^ software. During the measurement, the participants only wore a swimsuit and an acrylic swim cap [26]. Total body water and hydration level were assessed by means of bioelectric impedance, with Bodystat 1500 (Bodystat Inc., Douglas, UK), and via urine specific gravity measurement, with URYXXON^®^ Relax (Macherey-Nagel, Düren, Germany); results < 1.020 indicated proper hydration. During these bioimpedance analyses, recommended measurement conditions were strictly followed [27].

### 2.5. Exercise Tests

During each exercise session, all athletes performed two Wingate anaerobic tests interspersed with a dummy throw test.

Wrestling-specific performance capacity was measured using a specific dummy throw test, which was modified to reflect wrestling combat [28,29]. The test comprised two modes:

- slow mode—four compulsory *suplex* throws in 30 s
- quick mode—as many *suplex* throws as possible in 15 s

The modes in one round were performed alternately for 3 min. Each round consisted of four slow mode parts and four quick mode parts. The score of the test was the number of properly executed throws only performed in the quick mode parts. Before the beginning of the test, athletes were given all necessary instructions. The size of the dummy was adjusted to the body weight and height of each athlete. 

Anaerobic capacity was assessed using the classical Wingate test on a cycloergometer (Monark 894E, Varberg, Sweden), following the recommendations for such tests as proposed by Bar-Or [30]. The Wingate test was performed twice; the first (WT_1_) 5 min before and the second (WT_2_) 10 min after the dummy throw (DT) test (Figure 1). The seat height was adjusted to each participant’s satisfaction and toe clips with straps were used to prevent the feet from slipping off the pedals. The primary test was preceded by a 5-min warm-up period of approximately 50 W power. This was followed by two run-up practices of 3 s, during which the actual test load was imposed to make the participants accustomed to the resistance. The test lasted for 30 s. External loading was estimated individually at 7.5% body weight. During the test, the athletes were encouraged to exert maximum effort. The recorded results included the peak power output, the average power output, the minimal power output, the time-to-peak power, and the maximum speed, which were analyzed using the Monark Anaerobic Test Software (ver. 3.0.1, 2009, Monark, Varberg, Sweden). 

### 2.6. Blood Samples Analysis

Fingertip blood samples were taken immediately pre-exercise (before the first Wingate test) and 3 min post-exercise (after the second Wingate test). All blood samples were taken with the patient in a seated upright position. Blood samples were immediately transferred to microtubes containing 500 µL of 0.6 M perchloric acid. Glucose concentration was measured using a colorimetric enzymatic method with glucose oxidase (Liquick Cor-GLUCOSE, Cormay, Łomianki, Poland). Lactate concentration measurements were taken according to the method described by Maughan [31]. All biochemical analyses were conducted using a Synergy 2 SIAFRT microplate multi-detection reader (BioTek, Winooski, VT, USA).

### 2.7. Gastrointestinal Side Effects

On day 1, 3, 5, 8, and 10 of supplementation, the participants completed a validated questionnaire to measure gastrointestinal side effects [32]. The adapted questionnaire consisted of 19 items describing common gastrointestinal symptoms. The numeric rating scale (NRS) (0–10 scale, with zero reflecting no gastrointestinal distress and 10 indicating the most severe gastrointestinal distress), was used to rate the intensity of those symptoms because NRS is a valid and reliable tool for measuring patient-assessed global pain [33].

### 2.8. Statistical Analysis

First, the normality of data was tested using the Shapiro-Wilk test. If the distribution was not normal, a Box-Cox transformation was applied. Repeated-measures analysis of variance (ANOVA) was used for the dummy throw test. For the Wingate test (performed twice on each testing day before and after the dummy throw test) and blood samples (taken before and after exercise) analysis, the double-multivariate repeated-measures ANOVA was used. The data were analyzed using the software program STATISTICA-12 (StatSoft Inc., Tulsa, OK, USA). The two factors were as follows: Wingate test 1—Wingate test 2 (first factor) within pre-intervention—post-intervention (second factor). This analysis incorporated the R-side covariation structure. The analysis also included factors independent of time: gender (male/female), treatment (sodium bicarbonate/placebo), and all double interactions. To eliminate gender interaction, the following triple interactions were also included: gender x treatment x period and gender x dummy throw test x treatment. Effect size was calculated as Cohen’s *ƒ*^2^; as follows: *f*^2^ = η^2^/(1 − η^2^).

Repeated-measures ANOVA required a total sample size of 46 individuals to achieve 0.95 power with an α of 0.05 for the two groups and four repeated measures (for the time-to-peak power). The average correlation between the repeated measures was 0.36 and for the medium effect size, partial η = 0.06 (both based on our pilot study in this population). The power calculations were performed using the software G*Power 3 (Dusseldorf, Germany).

## 3. Results

### 3.1. Gastrointestinal Side Effects

No gastrointestinal side effects (*p* > 0.05) were reported by the participants throughout the study (Table 2). 

### 3.2. Anaerobic Power in the Wingate Test

**Figure 2:**
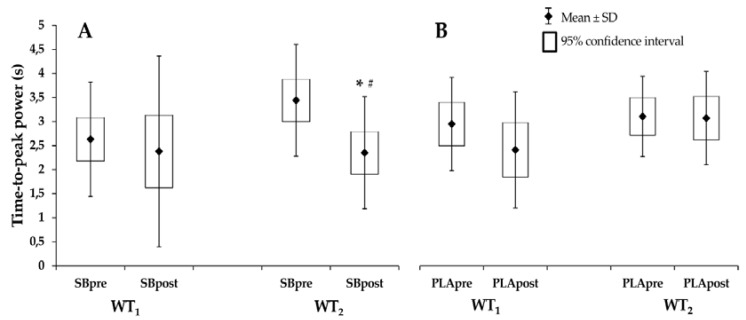
Time-to-peak power in the anaerobic Wingate test. (**A**) Before and after sodium bicarbonate. (**B**) Before and after placebo. Values are means ± standard deviation (and 95% confidence intervals). Abbreviations: SBpre—before sodium bicarbonate supplementation, SBpost—after sodium bicarbonate supplementation, PLApre—before placebo treatment, PLApost—after placebo treatment, WT_1_—the Wingate test before the dummy throw test, WT_2_—the Wingate test after the dummy throw test. * significantly different from SB_PRE_ in the WT_2_ (*p* = 0.0018), **^#^** significantly different from PLA_POST_ in the WT_2_ (*p* = 0.0142).

On each testing day, the Wingate test was performed twice; before and after the dummy throw test. Only the time-to-peak power was significantly different following sodium bicarbonate administration (*f*^2^ = 4%). The time-to-peak power in the second Wingate test decreased significantly (*p* = 0.0018) in the SB group from 3.44 ± 1.98 s to 2.35 ± 1.17 s (Figure 2). The time-to-peak power in the second Wingate test was also significantly more improved in the SB group than in the PLA group (SB group after exercise: 2.35 ± 1.17 s vs. PLA group after exercise: 3.08 ± 0.97 s; *p* = 0.0142) (Figure 2).

No significant differences were found between sodium bicarbonate and placebo in peak power (7.3% difference in WT_1_; 9.6% difference in WT_2_), average power (4.2% difference in WT_1_; 5.9% difference in WT_2_), and minimum power (6.1% difference in WT_1_; 2.7% difference in WT_2_) (Table 3). After treatments, in the SB group, there was a nonsignificant increase in peak power (increase of 8.3% in WT_1_; increase of 12% in WT_2_) and average power (increase of 0.9% in WT_1_; increase of 5.0% in WT_2_). Minimum power decreased non-significantly in the SB group by 2.7% in WT_1_, but it was unchanged in WT_2_.

### 3.3. Dummy Throw Test

**Figure 3:**
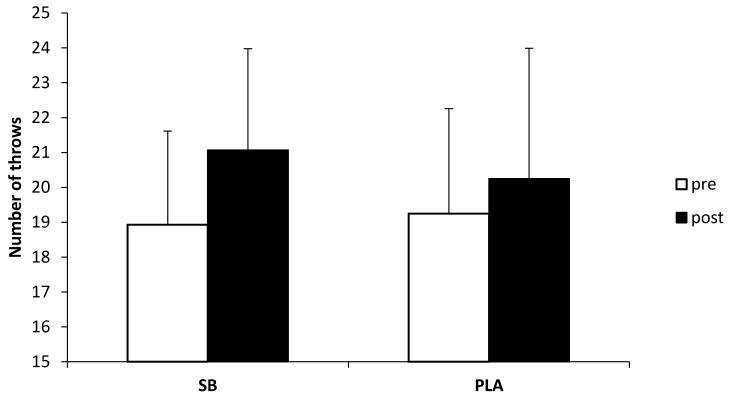
Total number of throws in the dummy throw test. SB—sodium bicarbonate group; PLA—placebo group.

No significant differences were found in dummy throws number (*p* = 0.0711, *f*^2^ = 4%). Before sodium bicarbonate administration, the total number of throws performed during the quick modes was on average 18.9 ± 2.7, whereas, after sodium bicarbonate administration, the number increased by two throws (average 21.1 ± 2.9) (Figure 3). On placebo administration, the number of throws only increased by one, from 19.3 ± 3.0 throws to 20.3 ± 3.7 throws; the differences in the number of throws was not statistically significant. 

### 3.4. Glucose and Lactate Concentrations

The glucose and lactate concentrations are given in Table 4. No significant differences were found in lactate and glucose concentrations.

## 4. Discussion

In our study, athletes were supplemented with sodium bicarbonate or placebo for 10 days in order to examine the effect of a new low-dose sodium bicarbonate loading regimen. We did not observe any significant changes in the SB and PLA groups regarding peak, average, or minimum power in the anaerobic Wingate test after the intervention. However, the time-to-peak power in the second Wingate test decreased significantly with sodium bicarbonate. The performance in the dummy throw test was unchanged following the supplementation. In addition, there were no changes in blood lactate or glucose concentrations.

The main limitation to sodium bicarbonate use in sports is its gastrointestinal side effects. In a previous study, after acute sodium bicarbonate intake, all participants reported gastrointestinal distress [24]. The most common symptoms included diarrhea, thirst, and bloating. This effect usually appears when sodium bicarbonate is ingested at the typically recommended dose of 300 mg·kg^−1^ [24]. In a study by Saunders et al. [25], the improvement in exercise capacity was only observed when participants experiencing gastrointestinal discomfort after sodium bicarbonate ingestion were excluded from the analysis. Because of the risk of gastrointestinal discomfort, sodium bicarbonate intake is infrequent, even in populations that would benefit from it the most. Thus, it is necessary to identify protocols that alleviate the gastrointestinal symptoms of sodium bicarbonate. In our study, sodium bicarbonate was supplemented in a multiday progressive-dose regimen. The supplementation started at a dose of 25 mg·kg^−1^ and was gradually increased to 50 mg·kg^−1^, 75 mg·kg^−1^, and finally to 100 mg·kg^−1^ daily, so that the greatest dose was three times smaller than in previous studies [24,25]. This new protocol allowed us to eliminate gastrointestinal symptoms through the gradual adjustment. 

However, the results obtained in our study seem to indicate that the supplemented doses of sodium bicarbonate were too small to elicit significant improvements in anaerobic capacity and wrestling performance. Nevertheless, we would like to highlight that a trend appeared towards increased peak, average, and minimum power in both anaerobic Wingate tests (before and after the dummy throw test), although the differences were not statistically significant. Despite this, the peak power increased by ~8% in WT_1_ and by ~12% in WT_2_ after SB supplementation compared to the baseline. The peak power after sodium bicarbonate treatment was higher than that after placebo treatment by ~7% and ~10% for WT_1_ and WT_2_, respectively, but the differences were not statistically significant. Only the time-to-peak power in the second Wingate test (after the dummy throw test) was significantly shorter (by ~1.1 s, i.e., ~32%) after sodium bicarbonate treatment.

These results are in agreement with a study by Zabala et al. [34], in which sodium bicarbonate ingestion had no effect on performance during a series of three Wingate tests interspersed by 30 min of recovery. The authors suggested that the short duration of the Wingate test (30 s) and the long recovery time interspersed between efforts (30 min) may explain the lack of an ergogenic benefit [34]. In contrast, in another study, sodium bicarbonate ingestion improved peak (+8%) and average (+2.9%) mechanical power, as well as the total work done (+3.9%) during six 10-s sprints on a cycloergometer, interspersed by 1 min of active recovery [35]. The inconsistency between those two studies might result from the different exercise testing protocols (30-s vs. 10-s sprints; 30-min vs. 1-min recovery). Other studies used upper body Wingate testing. Artioli et al. [14] assessed the effect of sodium bicarbonate on four bouts of 30-s upper body Wingate tests. The average and peak power only improved in the final bouts (average power: in bout 3 and 4, peak power: in bout 4). Based on the results, the authors suggested that the ergogenic effect of sodium bicarbonate might be particularly apparent after the onset of fatigue [14]. In a study by Tobias et al. [36], in order to evaluate the effect of seven-day sodium bicarbonate supplementation on exercise performance, athletes completed a four-bout upper-body Wingate test. Each bout lasted 30 s and the load was set at 5% of the body weight. The total work done was enhanced by 8% after sodium bicarbonate treatment. Moreover, a significant increase in mean power (+9.4%, *p* = 0.038) was only observed in bout 4, and approached a significant effect in bouts 2 (*p* = 0.093) and 3 (*p* = 0.079) [36]. Peak power likewise only improved in bout 4 (+13.7%, *p* = 0.018), and approached a significant improvement in bout 1 (*p* < 0.1) in that study [36]. A similar exercise protocol (four 30-s Wingate bouts for upper body with 3-min recovery between bouts) was used by Olivera et al. [37], who observed a significant difference in the total mechanical work done (+2.86%, *p* = 0.02) between sodium bicarbonate and placebo supplementation for five days, especially in the last two bouts (bouts 3 + 4: +5.93%, *p* = 0.02) of sprints. 

In our study, the time-to-peak power was improved in the second Wingate test, after the dummy throw test, which suggests that the greatest effect of sodium bicarbonate supplementation is observed in the late stages of high-intensity exercise. This was observed in the context of peak and average power, as well as in the total mechanical work done in previous studies [14,37]. The lack of improvement in power parameters in our study was plausibly due to the small dose of sodium bicarbonate. Compared to previous studies which used 300 to 500 mg·kg^−1^ of sodium bicarbonate [14,34,35,36,37], the participants in our study only ingested up to 100 mg·kg^−1^. In fact, McNaughton did not observe ergogenic efficacy of the acute ingestion of sodium bicarbonate in a dose of 100 mg·kg^−1^ BM [38]. However, in another study, McKenzie et al. [39] did not reveal significant differences in total work done and performance time between a sodium bicarbonate dose of 0.15 g·kg^−1^ BM and 0.3 g·kg^−1^ BM. As mentioned before, Browman [23] observed a favorable effect of an acute dose of 100 mg·kg^−1^ BM sodium bicarbonate on performance in swimmers. Of note, in all studies mentioned above, the acute protocol of sodium bicarbonate intake was applied. It has to be mentioned that acute and chronic intake may elicit different responses. For instance, Mc Naughton and Thompson [40] showed a more beneficial effect of chronic vs. acute bicarbonate intake of 0.5 g·kg^−1^ BM on the performance of 90 s maximal cycling ergometry. On the contrary, Carr et al. [41] and Joyce et al. [42] did not find any beneficial effect of the chronic over acute sodium bicarbonate supplementation in 2000 m rowing performance and in 200 m swim performance. 

We observed a decrease in time to peak power in the second Wingate test, which means that athletes were able to produce peak power faster. This would be especially beneficial in competitive combat. During the fatigue process, the reaction time (defined as the interval of time between presentation of a non-anticipated stimuli and the onset of response), response time (described as the time required for performing a voluntary movement after a stimulus), and performance time (defined as the time interval between reaction time and response time) may affect an athlete’s capacity to attack, defend, escape, or respond effectively to an opponent’s action [43,44]. It is well known that in competitive combat, the success during a match is often determined by fast actions performed after a stimulus and that it can be influenced by fatigue [45,46]. Decreasing the time of producing peak power may contribute to better performance in a match and possibly decide on the victory.

To our knowledge, this is the first study to measure the effect of chronic sodium bicarbonate supplemented according to the small dose protocol on wrestling-specific performance. Previous studies on combat athletes focused on boxing, taekwondo, and judo [14,15,16,47]. For instance, in a study on elite boxers, sodium bicarbonate increased the total punch efficacy (+5.4%) during a boxing match [16]. Similarly, during a simulated taekwondo combat, the summed attack time in three rounds was significantly higher (+13%) in the sodium bicarbonate group than in the placebo group [47]. Performance enhancement after sodium bicarbonate intervention was also reported in judo athletes. The total number of throws in three bouts of SJFT was higher by four throws in sodium bicarbonate therapy than in placebo therapy [14]. However, when each round was analyzed separately, significant differences were only observed in bouts 2 (27.4 throws for sodium bicarbonate vs. 25.9 throws for placebo) and 3 (27.0 throws for sodium bicarbonate vs. 25.6 throws for placebo). In another study, the total number of throws performed in three bouts of SJFT was not significantly different between the sodium bicarbonate and placebo groups [15]. Only in bout 3 was the number of throws significantly higher for sodium bicarbonate than for the placebo (23.7 ± 1.1 throws vs. 22.5 ± 1.6 throws). These results highlight the importance of a buffering potential at the end of high-intensity exercise when acidosis increases. Therefore, it is plausible that the ergogenic effect of sodium bicarbonate would have been observed in our study if another session of the dummy throw test had been introduced. However, in our opinion, another 3-min dummy throw test would have been overly exhaustive and might have endangered the athletes’ health.

In our study, there were also no changes in lactate or glucose concentration. In contrast, in previous studies, sodium bicarbonate ingestion led to increased blood lactate concentrations [14,15,47]. Elevated lactate concentrations might be a result of increased lactate and H^+^ efflux from muscle cells [48,49]. This allows for the maintenance of muscle cell pH within the physiological range necessary for muscle contractility [8]. A meta-analysis by Carr et al. [18] suggested that a reference dose of sodium bicarbonate (3.5 mmol∙kg^−1^) may result in a moderate performance enhancement, while a small extra increase in this performance effect is possible with an increase in dose to 4.5 mmol∙kg^−1^. Furthermore, although individual responses may vary, these authors recommend ingestion of 300- to 500 mg·kg^−1^ sodium bicarbonate to improve mean power in high-intensity races of short duration [18]. This indicates that the doses in our study could have been too small to result in increased lactate and H^+^ efflux and enhanced performance.

Although the doses of sodium bicarbonate used in our study were relatively small, which might explain the lack of statistically significant differences, we reported a trend towards improved anaerobic capacity and wrestling performance and a significant improvement in the second Wingate test. In elite sports, even small improvements may influence the chances of winning a competition. Therefore, as this new sodium bicarbonate supplementation regimen did not cause any side effects and tended to improve performance, it might be beneficial to wrestlers. Moreover, in our study, the participants were elite athletes competing at the highest level. It is hard to induce spectacular performance improvements in elite athletes already at the best of their physical capacities. Nevertheless, further research should focus on confirming our results using a progressive sodium bicarbonate-loading regimen with higher doses.

## 5. Conclusions

Our study showed that although our new loading sodium bicarbonate regimen eliminated gastrointestinal side effects, the doses used might have been too small and/or not sufficiently adjusted individually to elicit improvements in anaerobic capacity and wrestling performance. Only the time-to-peak power in the second Wingate test improved significantly after sodium bicarbonate supplementation, but the effect size was rather small. In practice, however, this can determine the final success of an athlete competing in the state of fatigue in combat sports.

## Figures and Tables

**Table 1:** Participants’ characteristics.

Variable	Sodium Bicarbonate Group	Placebo Group
	Mean ± SD	Mean ± SD
Number of subjects (*n*)	29	20
Age (years)	19 ± 4	18 ± 4
Height (cm)	173 ± 9	171 ± 7
Training types [the number of training sessions during one week]:		
Wrestling training	5 ± 1	5 ± 1
Running training	1 ± 1	2 ± 1
Resistance training	1 ± 1	1 ± 0

**Table 2:** Gastrointestinal side effects during sodium bicarbonate and placebo supplementation.

Variable	Sodium Bicarbonate Trial					Placebo Trial				
	Day 1	Day 3	Day 5	Day 8	Day 10	Day 1	Day 3	Day 5	Day 8	Day 10
**Stomach problems**	1.0 ± 1.0	0.8 ± 0.9	0.6 ± 1.0	0.5 ± 0.8	0.3 ± 0.6	0.8 ± 1.2	0.3 ± 0.6	0.6 ± 0.8	0.8 ± 1.0	0.6 ± 0.9
**Nausea**	0.3 ± 0.6	0.4 ± 0.6	0.2 ± 0.6	0.2 ± 0.5	0.1 ± 0.4	0.3 ± 0.5	0.3 ± 0.7	0.3 ± 0.4	0.3 ± 0.6	0.2 ± 0.5
**Dizziness**	0.1 ± 0.3	0.1 ± 0.4	0.3 ± 0.8	0.1 ± 0.3	0.1 ± 0.3	0.2 ± 0.4	0.2 ± 0.7	0.3 ± 0.4	0.1 ± 0.3	0.0 ± 0.0
**Headache**	0.6 ± 0.5	0.6 ± 0.9	0.2 ± 0.8	0.2 ± 0.6	0.4 ± 0.8	0.5 ± 0.9	0.6 ± 1.0	0.7 ± 1.0	0.4 ± 0.8	0.3 ± 0.8
**Flatulence**	0.3 ± 0.6	0.2 ± 0.4	0.3 ± 0.5	0.2 ± 0.4	0.2 ± 0.4	0.2 ± 0.5	0.2 ± 0.5	0.2 ± 0.4	0.1 ± 0.3	0.1 ± 0.3
**Urge to urinate**	0.3 ± 0.6	0.2 ± 0.5	0.3 ± 0.5	0.1 ± 0.4	0.1 ± 0.3	0.3 ± 0.6	0.2 ± 0.4	0.4 ± 0.6	0.2 ± 0.4	0.2 ± 0.4
**Urge to defecate**	0.3 ± 0.7	0.2 ± 0.5	0.1 ± 0.6	0.4 ± 0.8	0.4 ± 0.8	0.3 ± 0.6	0.3 ± 0.7	0.6 ± 1.0	0.3 ± 0.6	0.2 ± 0.4
**Belching**	0.4 ± 0.9	0.3 ± 0.7	0.2 ± 0.5	0.3 ± 0.7	0.2 ± 0.5	0.1 ± 0.3	0.3 ± 0.7	0.2 ± 0.4	0.3 ± 0.7	0.2 ± 0.5
**Heartburn**	0.3 ± 0.7	0.2 ± 0.6	0.2 ± 0.6	0.1 ± 0.4	0.2 ± 0.6	0.1 ± 0.3	0.0 ± 0.0	0.1 ± 0.4	0.3 ± 0.6	0.2 ± 0.4
**Bloating**	0.2 ± 0.6	0.2 ± 0.6	0.3 ± 0.7	0.4 ± 0.8	0.3 ± 0.8	0.4 ± 0.9	0.2 ± 0.4	0.4 ± 0.8	0.3 ± 0.6	0.5 ± 0.8
**Stomach cramps**	0.0 ± 0.0	0.1 ± 0.4	0.3 ± 0.6	0.3 ± 0.8	0.3 ± 0.9	0.3 ± 0.5	0.3 ± 0.7	0.4 ± 0.7	0.4 ± 0.7	0.1 ± 0.3
**Intestinal cramps**	0.2 ± 0.7	0.2 ± 0.4	0.1 ± 0.3	0.1 ± 0.3	0.2 ± 0.5	0.2 ± 0.5	0.6 ± 0.9	0.1 ± 0.3	0.1 ± 0.3	0.1 ± 0.2
**Urge to vomit**	0.0 ± 0.0	0.1 ± 0.4	0.0 ± 0.2	0.0 ± 0.2	0.0 ± 0.2	0.0 ± 0.0	0.1 ± 0.3	0.3 ± 0.6	0.1 ± 0.2	0.1 ± 0.3
**Vomiting**	0.0 ± 0.0	0.0 ± 0.0	0.0 ± 0.2	0.0 ± 0.0	0.0 ± 0.0	0.0 ± 0.0	0.3 ± 0.8	0.1 ± 0.2	0.0 ± 0.0	0.0 ± 0.0
**Diarrhea**	0.2 ± 0.5	0.2 ± 0.5	0.2 ± 0.6	0.3 ± 0.7	0.2 ± 0.4	0.5 ± 0.9	0.0 ± 0.0	0.2 ± 0.4	0.2 ± 0.6	0.3 ± 0.7
**Side ache left**	0.2 ± 0.5	0.0 ± 0.2	0.0 ± 0.2	0.0 ± 0.2	0.0 ± 0.2	0.0 ± 0.0	0.5 ± 0.8	0.4 ± 0.7	0.1 ± 0.2	0.1 ± 0.2
**Side ache right**	0.0 ± 0.2	0.0 ± 0.2	0.0 ± 0.2	0.0 ± 0.0	0.0 ± 0.2	0.0 ± 0.0	0.2 ± 0.4	0.0 ± 0.0	0.0 ± 0.0	0.2 ± 0.5
**Muscle cramps**	0.1 ± 0.3	0.3 ± 0.7	0.1 ± 0.4	0.3 ± 0.6	0.2 ± 0.6	0.6 ± 1.0	0.3 ± 0.6	0.2 ± 0.6	0.2 ± 0.6	0.1 ± 0.3
**Cold shivering**	0.1 ± 0.6	0.2 ± 0.7	0.1 ± 0.3	0.0 ± 0.0	0.0 ± 0.0	0.3 ± 0.7	0.0 ± 0.0	0.0 ± 0.0	0.0 ± 0.0	0.1 ± 0.2

Data are mean ± standard deviation.

**Table 3:** Power Indices in the Wingate Anaerobic Test.

Variable		*p-Value* Anova (Effect Size *f*^2^*%*)	*p-Value* (SB_PRE_ vs. PLA_PRE_)	SB_PRE_	SB_POST_	*p-Value* (SB_PRE_ vs. SB_POST_)	PLA_PRE_	PLA_POST_	*p-Value* (PLA_PRE_ vs. PLA_POST_)	*p-Value* (SB_POST_ vs. PLA_POST_)
**Peak power (W)**	WT_1_	0.6021 (0.5%)	1.0000	834 ± 316 (714–954)	903 ± 330 (777–1028)	0.1826	802 ± 310 (657–947)	837 ± 288 (702–972)	1.0000	1.0000
	WT_2_		1.0000	791 ± 173 (678–903)	886 ± 172 (769–1003)	0.0032	751 ± 147 (631–870)	801 ± 142 (676–927)	1.0000	1.0000
**Average power (W)**	WT_1_	0.0862 (2.6%)	1.0000	548 ± 102 (482–613)	553 ± 108 (487–618)	1.0000	526 ± 80 (457–595)	530 ± 90 (464–596)	1.0000	1.0000
	WT_2_		1.0000	517 ± 296 (452–582)	543 ± 309 (479–607)	0.0097	507 ± 255 (440–574)	511 ± 268 (444–579)	1.0000	1.0000
**Minimum power (W)**	WT_1_	0.3575 (1.1%)	1.0000	336 ± 171 (297–375)	327 ± 168 (286–368)	1.0000	319 ± 143 (282–357)	307 ± 144 (265–349)	1.0000	1.0000
	WT_2_		1.0000	297 ± 97 (260–334)	298 ± 110 (256–340)	1.0000	301 ± 87 (261–342)	290 ± 85 (250–330)	1.0000	1.0000

Values are mean ± SD (and 95% confidence intervals). Abbreviations: SBpre—before sodium bicarbonate supplementation, SBpost—after sodium bicarbonate supplementation, PLApre—before placebo treatment, PLApost—after placebo treatment, WT_1_—the Wingate test before the dummy throw test, WT_2_—the Wingate test after the dummy throw test.

**Table 4:** Glucose and lactate concentrations.

		*p-*Value Anova (Effect Size *f*^2^*%*)	*p-*Value (SB_PRE_ vs. PLA_PRE_)	SB_PRE_	SB_POST_	*p-*Value (SB_PRE_ vs. SB_POST_)	PLA_PRE_	PLA_POST_	*p-*Value (PLA_PRE_ vs. PLA_POST_)	*p-*Value (SB_POST_ vs. PLA_POST_)
**Glucose (mg∙dL^−1^)**	**Pre-exercise**	0.4848 (0.7%)	1.0000	118.3 ± 16.6 (110.3–125.9)	102.6 ± 20.5 (96.3–115.5)	0.1060	124.2 ± 19.9 (114.9–133.6)	113.0 ± 14.9 (106.0–119.9)	1.0000	1.0000
	**Post-exercise**		1.0000	139.3 ± 24.0 (125.6–146.9)	130.9 ± 25.7 (109.1–133.8)	1.0000	135.0 ± 24.1 (123.7–146.3)	131.4 ± 22.5 (120.9–142.0)	1.0000	1.0000
**Lactate (mmol∙L^−1^)**	**Pre-exercise**	0.2044 (1.4%)	1.0000	1.56 ± 0.42 (1.40–1.73)	1.51 ± 0.40 (1.30–1.75)	1.0000	1.68 ± 0.46 (1.56–2.40)	1.67 ± 0.57 (1.44–2.19)	1.0000	1.0000
	**Post-exercise**		1.0000	16.37 ± 2.25 (15.57–17.62)	16.82 ± 2.82 (13.72–16.98)	1.0000	15.98 ± 2.73 (14.58–17.19)	15.77 ± 2.65 (14.12–17.02)	1.0000	1.0000

Values are means ± SD (and 95% confidence intervals). Abbreviations: SBpre—before sodium bicarbonate supplementation, SBpost—after sodium bicarbonate supplementation, PLApre—before placebo treatment, PLApost—after placebo treatment.

## Supplementary Materials

The following are available online at http://www.mdpi.com/2072-6643/10/6/697/s1. Table S1: CONSORT 2010 checklist of information to include when reporting a randomised trial.
